# Lack of association between *G6PD* variants and Parkinson disease

**DOI:** 10.1016/j.xhgg.2025.100555

**Published:** 2025-12-09

**Authors:** Leah V. Chifamba, Sitki Cem Parlar, Lang Liu, Leonard L. Sokol, Eric Yu, Farnaz Asayesh, Jamil Ahmad, Jennifer A. Ruskey, Dan Spiegelman, Cheryl Waters, Oury Monchi, Yves Dauvilliers, Nicolas Dupré, Alla Timofeeva, Anton Emelyanov, Sofya Pchelina, Irina Miliukhina, Lior Greenbaum, Sharon Hassin-Baer, Roy N. Alcalay, Alberto J. Espay, Ziv Gan-Or, Konstantin Senkevich

**Affiliations:** 1 The Neuro (Montreal Neurological Institute-Hospital), McGill University, Montreal, QC, Canada; 2 Department of Human Genetics, McGill University, Montréal, QC, Canada; 3 Department of Molecular Medicine and Division of Neurology, Department of Medicine, Scripps, La Jolla, CA, USA; 4 Department of Neurology and Neurosurgery, McGill University, Montréal, QC, Canada; 5 Department of Neurology, College of Physicians and Surgeons, Columbia University Medical Center, New York, NY, USA; 6 Centre de recherche de l’Institut universitaire de gériatrie de Montréal, Montréal, QC, Canada; 7 Département de radiologie, radio-oncologie et médecine nucléaire, Université de Montréal, Montréal, QC, Canada; 8 National Reference Center for Narcolepsy, Sleep Unit, Department of Neurology, Guide-Chauliac Hospital, CHU Montpellier, University of Montpellier, Montpellier, France; 9 Neuroscience Axis, CHU de Québec-Université Laval, Québec, QC, Canada; 10 Department of Medicine, Faculty of Medicine, Université Laval, Quebec City, QC, Canada; 11 Pavlov First State Medical University of St. Petersburg, St. Petersburg, Russia; 12 Institute of the Human Brain of RAS, St. Petersburg, Russia; 13 Danek Gertner Institute of Human Genetics, Sheba Medical Center, Tel Hashomer, Ramat Gan, Israel; 14 Tel Aviv School of Medicine, Tel Aviv University, Tel Aviv, Israel; 15 Movement Disorders Institute, Department of Neurology, Sheba Medical Center, Tel Hashomer, Israel; 16 Division of Movement Disorders, Tel Aviv Sourasky Medical Center, Tel Aviv, Israel; 17 Faculty of Medical & Health Sciences, Tel Aviv University, Tel Aviv, Israel; 18 James J. and Joan A. Gardner Family Center for Parkinson’s Disease and Movement Disorders, University of Cincinnati, Cincinnati, OH, USA; 19 Department of Specialized Medicine, Division of Medical Genetics, McGill University Health Centre, Montreal, QC, Canada

**Keywords:** Parkinson’s disease, G6PD, oxidative stress, X-linked genetics, rare variant analysis, common variant association, SKAT-O

## Abstract

Oxidative stress has been implicated in Parkinson disease (PD). Genes involved in PD, such as *PRKN*, *PINK1*, and *PARK7*, contribute to oxidative stress in dopaminergic neurons. The X-linked G6PD gene encodes glucose 6-phosphate dehydrogenase, an important regulator of oxidative stress. Recent studies suggested that alpha-synuclein aggregates may impair G6PD activity and contribute to dopaminergic neuron loss, and that *G6PD* mutations may independently increase the risk of PD. In this study, we aimed to examine the role of common and rare *G6PD* variants in PD across 6 cohorts, including 8,905 PD cases, 16,770 proxy cases, and 394,098 controls. These cohorts were analyzed after stratification by sex and then combined to account for the *G6PD* X-linked location. Using logistic regression, we did not identify significant associations for common variants in any of the cohorts. The optimized sequence Kernel association (SKAT-O) test was performed to assess the effect of rare variants (minor allele frequency <0.01) across six cohorts, followed by a meta-analysis using metaSKAT, also demonstrating lack of association. In conclusion, we did not find evidence for a role for *G6PD* in PD.

## Teaser

We investigated common and rare variants in the X-linked G6PD gene across six large cohorts of Parkinson disease patients and controls. Our results show no significant associations, suggesting G6PD is unlikely to play a major role in Parkinson disease risk.

## Introduction

Oxidative stress is one of the factors playing a role in the pathogenesis of Parkinson disease (PD).^1^ Core genes implicated in the PD pathway, such as *PRKN*, *PINK1*, and *PARK7*, contribute to oxidative stress, impacting dopaminergic neurons.^2^

Glucose 6-phosphate dehydrogenase (*G6PD*) is an X-linked gene that encodes for the G6PD enzyme, which regulates oxidative stress.^3^ The enzyme is a critical component of the pentose phosphate pathway, where it catalyzes the production of nicotinamide adenine dinucleotide phosphate (NADPH), thereby preventing cellular damage.^4^ The role of G6PD has been investigated mostly in hemolytic anemia, where G6PD deficiency leads to oxidative damage in erythrocytes.^5^ While G6PD deficiency predominantly affects males,^6^ it has also manifested in females carrying two mutated copies of the gene.^7^

A recent study suggested that alpha-synuclein aggregates, a feature of most PD cases, may lead to loss of G6PD within synaptic vesicles, resulting in decreased NADPH and oxidative damage in dopaminergic neurons.^8^ The authors also suggested a genetic association between *G6PD* missense mutations and PD.^8^ *G6PD* has not been identified as associated with PD in previous X-wide association studies (XWASes), although it is located close to an associated locus.^9^ The nearest PD-associated SNP in the previous XWASes is rs28602900 (chrX:154,405,192), located ∼126 kb upstream of the G6PD (chrX:154,531,391–154,547,572). Linkage disequilibrium (LD) analysis using the 1000 Genomes European reference panel showed that rs28602900 is not in LD (*r*^*2*^ < 0.1) with *G6PD* common variants, and *G6PD* does not fall within the same LD block as this locus. Furthermore, another study has shown that deletion of *G6PD* using CRISPR-Cas9 impacted PINK1-Parkin-mediated mitophagy,^10^ a pathway involved in PD.^11^ This suggests that deficiency in G6PD may intensify mitochondrial dysfunction and oxidative stress, potentially contributing to the pathogenesis of PD.

Our study aimed to gain further understanding of the involvement of *G6PD* in PD by investigating the potential role of common and rare *G6PD* genetic variants in PD. The analysis included 6 independent cohorts with a total of 8,905 PD cases, 16,770 proxy cases, and 394,098 controls. The cohorts were analyzed after stratifying by sex and then combined to account for the *G6PD* X-linked location.

## Methods

### Study participants

The population for the genetic analysis comprised 6 cohorts, 8,905 PD cases, 16,770 proxy cases, and 394,098 controls (Table 1). The patients were diagnosed based on movement disorders specialist of the UK Biobank (UKBB) Brain Bank Criteria,^12^ or Movement Disorders Society Criteria.^13^ The first four cohorts were collected at McGill University and have been previously reported. In brief, they include a (1) French-Canadian/French cohort collected from Quebec, Canada^14^ and Montpellier, France; (2) Columbia University cohort, New York,^15^ (3) Sheba cohort from Sheba Medical Center, Israel,^16^ which comprised Ashkenazi and Mizrahi Jews; and (4) cohort from Pavlov First State Medical University and Institute of the Human Brain of the Russian Academy of Sciences Russia.^17^ Additional cohorts included (1) whole-genome sequencing (WGS) data from Accelerated Medicines Partnership-Parkinson’s Disease and Related Disorders (AMP-PD). The AMP-PD data included the Harvard Biomarkers Study, the Parkinson’s Progression Markers Initiative, the National Institute of Neurological Disorders and Stroke (NINDS) Parkinson’s Disease Biomarkers Program, the BioFIND study, the NINDS Study of Isradipine as a Disease Modifying Agent in Subjects With Early Parkinson’s Disease, phase 3, and the National Institute on Aging International Lewy Body Dementia Genetics Consortium Genome Sequencing in Lewy Body Dementia case-control cohort. (2) The UKBB was acquired using WGS data. UKBB phenotype data were derived from multiple fields, including International Classification of Diseases, 10^th^ Revision (ICD-10) diagnoses (field 41270), PD status (field 131023), genetic ethnic grouping (field 22006), and age at recruitment (field 21022). PD cases were defined as participants with a diagnosis of PD based on the relevant ICD or self-reported PD field. Analyses were conducted separately using two UKBB datasets—one including proxy cases and one excluding proxy cases—to assess potential effects of phenotype misclassification on the results. Proxy cases were defined as individuals with a parent or sibling affected by PD. Controls were without any reported nervous system disorders (category 2406), parental history of PD or dementia (fields 20107 and 20110), or neurological conditions such as dementia (42018), vascular dementia (42022), frontotemporal dementia (42024), amyotrophic lateral sclerosis (42028), parkinsonism (42030), PD (42032), progressive supranuclear palsy (42034), or multiple system atrophy (42036) was obtained from ICD-10 codes. Ethics approval for the research study was granted by the McGill University research ethics board.

**Table 1:** Demographics of studied cohorts

Cohort	N, cases	N, controls	Sex	N, cases	N, controls	Mean age (SD)	Mean age (SD)
McGill University	1,027	1,115	M	636	517	59.71 (11.41)	54.43 (14.33)
			F	391	598	60.39 (11.46)	54.25 (13.47)
Columbia University	1,070	492	M	691	172	59.38 (11.65)	67.11 (11.17)
			F	379	320	60.09 (11.89)	62.38 (10.04)
Pavlov First State Medical University and Institute of the Human Brain	469	332	M	182	113	61.40 (16.74)	68.48 (21.81)
			F	287	219	62.56 (17.41)	75.33 (14.58)
Sheba Medical Center	984	525	M	603	296	63.82 (12.58)	33.45 (8.93)
			F	381	229	64.58 (13.11)	33.31 (6.69)
AMP-PD	2,341	3,486	M	1,459	1,648	64.80 (9.82)	69.04 (13.58)
			F	882	1,838	63.29 (10.04)	67.73 (13.45)
UKBB	2,966	64,936	M	1,873	30,006	63.11 (5.23)	56.98 (8.10)
			F	1,093	34,870	62.74 (5.29)	56.61 (7.93)
UKBB (with proxies)	19,736	387,955	M	9,007	178,331	59.57 (7.72)	57,03 (7.55)
			F	10,729	209,624	58.90 (7.65)	56.61 (7.52)

AMP-PD, Accelerated Medicines Partnership-Parkinson’s Disease and Related Disorders; SD, standard deviation; UKBB, UK Biobank.

### G6PD sequencing and quality control

Targeted next-generation sequencing of *G6PD* was performed using molecular inversion probes (MIPs) in the four cohorts gathered at McGill University as previously described.^18^ (The MIPs protocol is accessible at https://github.com/gan-orlab/MIP_protocol.) The Génome Québec Innovation Centre carried out the sequencing utilizing the Illumina NovaSeq 6000 SP PE100 platform. The Burrows-Wheeler Aligner (hg19) was used for alignment,^19^ and the Genome Analysis Toolkit (GATK, version 3.8) was employed for post-alignment quality control and variant calling.^20^ Using PLINK program version 1.9,^21^ we carried out quality control by eliminating variants and samples of lower quality. SNPs with missingness of more than 10% were excluded from the analysis. Variants having a minimum quality score (GQ) of 30 and minimal depths of coverage 30× were included.

As previously described, quality control procedures for WGS for AMP-PD cohorts were carried out on an individual level and a variant level (https://amp-pd.org/whole-genome-data).^22^ We performed quality control on the UKBB WGS data using GATK version 3.8, using a minimum depth of coverage of 30× and GQ of 20 for further analysis. We applied hg38 reference for AMP-PD and UKBB.

### Statistical analyses

Power for common variants’ minor allele frequency (MAF) >1% was estimated with a case-control power calculator^23^ under a multiplicative model. For rare variants (MAF <1%), power was estimated with PAGEANT^24^ using Scenario S2, which assumes allele frequency is independent of per-allele effects. We specified locus-level explained variance (EV = 0.5%) and two-sided α = 0.05. Analyses were run considering all cohorts with and without UKBB proxy cases. Since *G6PD* is located on the X chromosome, we stratified the cohorts by sex to account for differences in allele dosage, analyzing males and females separately. The results were then meta-analyzed across cohorts. To assess the association between common variants (MAF >0.01) in *G6PD* with PD, we conducted logistic regression, adjusting for age, using PLINK version 1.9.^21^ We also used the optimized sequence Kernel association (SKAT-O, R package)^25^ test to study the association of rare variants (MAF <0.01) with PD, and performed a meta-analysis using the metaSKAT package.^26^ Given the large number of UKBB controls and to mitigate bias from case-control imbalance, we randomly sampled a control subset at a 1:10 case:control ratio for both datasets (cases only and cases + proxies). These subsets were used for rare variant analyses, and results were meta-analyzed accordingly. We examined in each cohort the burden of five groups of variants: (1) all rare variants, (2) nonsynonymous variants, (3) functional variants (including stop/frameshift, splicing, and nonsynonymous variants), (4) variants with a high combined annotation-dependent deletion score ≥20, and (5) loss-of-function variants. Additionally, we included *G6PD* variants associated with mean enzyme activity of <20% of normal.^27^ These variants were selected based on the 2024 World Health Organization classification of *G6PD* variants, specifically Class A, which includes variants that significantly reduce enzyme activity and are associated with chronic hemolytic anemia.^28^ The description of these variants is summarized in Table S1. In all analyses, we controlled the false discovery rate (FDR). For common variants, we applied FDR across all identified common variants pooled across cohorts. Since analyses were stratified by sex, we additionally applied a Bonferroni correction across the three sex groups in each cohort. For SKAT-O test, we applied the FDR method using Benjamini-Hochberg.

## Results

We sequenced *G6PD* in four cohorts at McGill, achieving an average read depth of 1,068×, with >89% nucleotides covered at >30×. For common variants (MAF 5%, odds ratio [OR] 1.20, two-sided α = 0.005), power was >0.80 for analysis with and without proxy cases. For variants with MAF 1%, power was 0.39 in the group without proxies and 0.88 with proxies. For rare variants (PAGEANT, EV = 0.5%, α = 0.05), power exceeded 0.80 in both analyses to detect nominal significance. We discovered and analyzed nine common variants (MAF >1%) from all cohorts for their association with PD, none of which remained significant after multiple test correction (Table S2). We found 112 rare variants with MAF <1% in cohorts sequenced at McGill, 82 in AMP-PD, and 5,265 in the UKBB cohort (Table S3). Burden analysis before and after sex stratification using SKAT-O showed no association after meta-analysis in any of the variant categories including variants associated with reduced G6PD activity. Individual cohort results are summarized in Table S4.

We then analyzed the allele frequencies of the six rare *G6PD* missense variants reported by Stykel et al.^8^ in our cohorts (Table S5). p.Asp113Asn showed a nominal association with PD in UKBB with proxies subset (OR = 2.11, 95% confidence interval [CI] 1.10–4.20; *p* = 0.03). However, given its ultra-rare frequency (MAF cases + proxies = 1.78 × 10^−4^, controls = 4.28 × 10^−5^, gnomAD 4 × 10^−5^) and the lack of replication in UKBB without proxies (OR = 1.70, 95% CI 0.6–4.0; *p* = 0.27), this is unlikely to represent a true association. Rare variant burden analysis for these six missense variants did not yield significant association after FDR and meta-analysis (Table 2).

**Table 2:** Burden test of six G6PD variants^a^ previously linked to PD

Cohort	N cases	N controls	*p*	Pfdr (including UKBB with proxies)	Pfdr (including UKBB without proxies)
McGill University	1,027	1,115	0.390	0.584	0.780
Columbia University	1,070	492	0.696	0.695	0.695
Pavlov First State Medical University and Institute of the Human Brain	469	332	0.244	0.488	0.731
Sheba Medical Center	984	525	0.09	0.299	0.597
AMP-PD	2,341	3,486	0.528	0.633	0.731
UKBB with proxies	19,736	197,360	0.018	0.109	–
UKBB without proxies	2,966	29,660	0.600	–	0.792
Meta-analysis with UKBB (cases)	8,857	35,610	0.584	–	–
Meta-analysis with UKBB (cases + proxies)	25,627	203,310	1	–	–

Pfdr, false discovery rate adjusted p value.

a The six G6PD variants are rs137852318, X:154533067:A:T (hg38), X:154534437:G:A (hg38), rs1557229675, rs1050829, and rs5030870.

## Discussion

This study aimed to assess the association of common and rare *G6PD* variants with PD in six independent cohorts. None of the associations remained significant after multiple correction and did not reach significance in the meta-analysis. Overall, our findings suggest lack of genetic associations between common and rare *G6PD* variants with PD.

A recent study by Stykel,^8^ showed that alpha-synuclein aggregates may impair G6PD activity, contributing to dopamine loss. Using UKBB data from GeneBass,^29^ the authors suggested that *G6PD* variants may independently increase the risk of PD. However, our analysis of six independent cohorts does not support this association. We conducted an in-depth analysis stratified by sex to account for the X chromosome location of *G6PD*, an approach that was not implemented in the GeneBass study. Despite all our efforts, we did not find compelling genetic evidence for the involvement of this gene in PD.

To the best of our knowledge, parkinsonism has not been previously described in patients with G6PD deficiency, making a direct causative link between G6PD deficiency and PD unlikely. Some medications, including levodopa, have been identified to exacerbate anemia symptoms in patients with G6PD deficiency.^30^ Thus, current clinical evidence does not support a role for G6PD deficiency in PD.

Our study has several limitations. First, the study comprised mostly individuals of European ancestry, which limits genetic diversity. Second, different quality control procedures were used across cohorts, with AMP-PD processed through centralized Broad Institute pipelines and other cohorts filtered locally, likely contributing to fewer rare functional variants in AMP-PD.

In conclusion, our analyses showed a lack of association between *G6PD* common and rare variants with PD; therefore, future studies should further investigate the role of other oxidative stress related genes in PD.

## Data and code availability

All generated data, including the variants used in the analysis, are provided in the paper or the supplemental tables. The code is available at https://github.com/gan-orlab/G6PD-on-PD. The McGill cohorts are partially available through the Canadian Open Parkinson Network (C-OPN). Access, including genetic data, can be requested through the C-OPN data access committee (https://copn-rpco.ca/submit-a-request/). The AMP-PD data was assessed using the Terra platform https://amp-pd.org/. The UKBB was acquired using WGS data from the UKBB Research Analysis Platform (https://www.ukbiobank.ac.uk/).

## Acknowledgments

We would like to sincerely thank the participants from the various cohorts who contributed to this study. This research was partially funded by the Canada First Research Excellence Fund through McGill University’s Healthy Brains, Healthy Lives initiative, with additional support from Calcul Québec and Compute Canada. Z.G.-O. is supported by the Chercheurs-Boursiers Award from the Fonds de Recherche du Québec–Santé in collaboration with Parkinson Quebec and is a William Dawson Scholar. Access to certain participants for this research was facilitated by the Quebec Parkinson’s Network (http://rpq-qpn.ca/en/). Access to UKBB data was supported by the NeuroHub infrastructure under Application Number 45551. Data for this study were also sourced from the AMP-PD Knowledge Platform. More information about the study can be found at https://www.amp-pd.org. Detailed acknowledgments for the AMP-PD cohort can be found in Data S1.

## Declaration of interests

Z.G.-O. has received consultancy fees from Lysosomal Therapeutics, Idorsia, Prevail Therapeutics, Inceptions Sciences (now Ventus), Neuron23, Ono Therapeutics, Bial Biotech, Bial, Handl Therapeutics, UCB, Capsida, Denali, Simcere, Takeda Pharmaceuticals, Jazz Pharmaceuticals, EG427, Vanqua Bio, Lighthouse, Deerfield, and Guidepoint. A.J.E. has received grant support from the NIH and the Michael J. Fox Foundation for Parkinson’s Research; personal compensation as a consultant/scientific advisory board member for Mitsubishi Tanabe Pharma America (formerly Neuroderm), Amneal, Acorda, AbbVie, Bial, Kyowa Kirin, Supernus (formerly USWorldMeds), NeuroDiagnostics (SYNAPS Dx), Intrance Medical Systems, Merz, Praxis Precision Medicines, Citrus Health, and Herantis Pharma; compensation as Data Safety Monitoring Board chair of AskBio; and publishing royalties from Lippincott Williams & Wilkins, Cambridge University Press, and Springer. He is co-inventor of the patent “Compositions and methods for treatment and/or prophylaxis of proteinopathies.” He cofounded REGAIN Therapeutics to fund preclinical studies but relinquished the right to any personal income from future treatments.

## Supplemental information

Document S1. Tables S1–S5

Data S1. Supplemental acknowledgments

Document S2. Article plus supplemental information
