# Supplementary material for: Lack of association between G6PD variants and Parkinson disease
Source: HGG Adv. 2025 Dec 9;7(1):100555. doi: 10.1016/j.xhgg.2025.100555 (PMC12799763; doi:10.1016/j.xhgg.2025.100555)
Supplement: Document S1. Tables S1–S5 [file mmc1.pdf]

HGGA, Volume 7

Supplemental information

Lack of association between *G6PD* variants and Parkinson disease

Leah V. Chifamba, Sitki Cem Parlar, Lang Liu, Leonard L. Sokol, Eric Yu, Farnaz Asayesh, Jamil Ahmad, Jennifer A. Ruskey, Dan Spiegelman, Cheryl Waters, Oury Monchi, Yves Dauvilliers, Nicolas Dupré, Alla Timofeeva, Anton Emelyanov, Sofya Pchelina, Irina Miliukhina, Lior Greenbaum, Sharon Hassin-Baer, Roy N. Alcalay, Alberto J. Espay, Ziv Gan-Or, and Konstantin Senkevich

Acknowledgements

AMP-PD, a public-private partnership managed by the Foundation for the National Institutes of Health (FNIH), is funded by Celgene, GSK, the Michael J. Fox Foundation for Parkinson's Research, the National Institute of Neurological Disorders and Stroke, Pfizer, AbbVie, Sanofi, and Verily.

Genetic data for this research were obtained from several sources, including the Fox Investigation for New Discovery of Biomarkers (BioFIND), the Harvard Biomarker Study (HBS), the Parkinson's Progression Markers Initiative (PPMI), the Parkinson's Disease Biomarkers Program (PDBP), the International LBD Genomics Consortium (iLBDGC), and the STEADY-PD III Investigators. BioFIND is sponsored by The Michael J. Fox Foundation for Parkinson's Research (MJFF), with support from the National Institute for Neurological Disorders and Stroke (NINDS). The BioFIND Investigators were not involved in the review of data analysis or the manuscript content.

The HBS is a collaborative effort of investigators, with a full list available at https://www.bwhparkinsoncenter.org/biobank/, and is funded by philanthropy, NIH, and non-NIH sources. HBS investigators did not participate in the review of data or manuscript content. PPMI is a public-private partnership funded by the Michael J. Fox Foundation for Parkinson's Research and its partners, listed at www.ppmi-info.org/fundingpartners. PPMI investigators were also not involved in reviewing data or the manuscript content. More details on the study are available at www.ppmi-info.org. The PDBP consortium is supported by the NINDS at the NIH, and a full list of PDBP investigators can be found at https://pdbp.ninds.nih.gov/policy. The PDBP investigators did not review the data analysis or manuscript content.

The "Genome Sequencing in Lewy Body Dementia and Neurologically Healthy Controls: A Resource for the Research Community" dataset was created by the iLBDGC, co-directed by Dr. Bryan J. Traynor and Dr. Sonja W. Scholz of the NIH Intramural Research Program. The iLBDGC investigators did not participate in reviewing the data analysis or manuscript content. For a full list of contributions, refer to For a full list of contributions, refer to DOI: 10.1038/s41588-021-00785-3. STEADY-PD III is a 36-month, Phase 3, placebo-controlled trial assessing the efficacy of isradipine (10 mg daily) in 336 participants with early-stage Parkinson's disease. The trial was funded by NINDS and supported by the Michael J. Fox Foundation for Parkinson's Research and the Parkinson Study Group. The STEADY-PD III investigators did not review the data analysis or manuscript content. A full list of investigators can be found at https://clinicaltrials.gov/ct2/show/NCT02168842.
